# An approach to determining the most common causes of stillbirth in low and middle-income countries: A commentary

**DOI:** 10.12688/gatesopenres.14112.1

**Published:** 2023-07-04

**Authors:** Robert L. Goldenberg, Jaume Ordi, Dianna M. Blau, Natalia Rakislova, Vardendra Kulkarni, Najia Karim Ghanchi, Sarah Saleem, Shivaprasad S. Goudar, Norman Goco, Christina Paganelli, Elizabeth M. McClure, PURPOSe, CHAMPS, ISGlobal, and the MITS Surveillance Alliance investigators

**Affiliations:** 1 Obstetrics & Gynecology, Columbia University, New York, NY, USA; 2 ISGlobal, Universitat de Barcelona, Barcelona, Spain; 3 Center for Global Health, Centers for Disease Control and Prevention, Atlanta, Georgia, USA; 4 Department of Pathology, Bapuji Educational Association’s J.J.M. Medical College, Davangere, India; 5 Department of Pathology & Microbiology, Aga Khan University, Karachi, Pakistan; 6 Department of Community Health Sciences, Aga khan University, Karachi, Pakistan; 7 Women's and Children's Health Research Unit, KLE University, Belagavi, India; 8 Social, Statistical and Environmental Health Sciences, RTI International, Durham, NC, 27709, USA

**Keywords:** Stillbirth, cause of stillbirth, useful investigations, minimally invasive tissue sampling, pathology

## Abstract

Stillbirth, one of the most common adverse pregnancy outcomes, is especially prevalent in low and middle-income countries (LMICs). Understanding the causes of stillbirth is crucial to developing effective interventions. In this commentary, investigators working across several LMICs discuss the most useful investigations to determine causes of stillbirths in LMICs. Useful data were defined as 1) feasible to obtain accurately and 2) informative to determine or help eliminate a cause of death.

Recently, new tools for LMIC settings to determine cause of death in stillbirths, including minimally invasive tissue sampling (MITS) – a method using needle biopsies to obtain internal organ tissue from deceased fetuses for histology and pathogen identification in those tissues have become available. While placental histology has been available for some time, the development of the Amsterdam Criteria in 2016 has provided a useful framework to categorize placental lesions. The authors recommend focusing on the clinical history, the placental evaluation, the external examination of the fetus, and, when available, fetal tissue obtained by MITS, especially of the lung (focused on histology and microbiology) and brain/cerebral spinal fluid (CSF) and fetal blood (focused on microbiological analysis). The authors recognize that this approach may not identify some causes of stillbirth, including some genetic abnormalities and internal organ anomalies, but believe it will identify the most common causes of stillbirth, and most of the preventable causes.

## Disclaimer

The views expressed in this article are those of the author(s). Publication in Gates Open Research does not imply endorsement by the Gates Foundation.

Stillbirths are one of the most common adverse pregnancy outcomes in low and middle-income countries (LMIC). In some high-income countries, stillbirth rates of 2–3 per thousand births are seen, while in some LMICs, reported stillbirth rates are 10 to 15-fold higher and may range from 30 to 50 per 1000 births ^1, 2^. The Every Newborn Action Plan (ENAP) has set a goal for each country to have a stillbirth rate of <12/1000 births by 2030 ^3^. Many LMICs appear unlikely to achieve that goal.

In high-income countries, cause of death (COD) in stillbirths has been evaluated using several different methods, 35 by one count ^4^, but because of differences in methodologies, there is still little consensus about the major causes. There is even less consensus about causes of stillbirths in LMICs, in part because until recently, evaluating the causes of stillbirths or reducing stillbirths in those locations has not been a major priority ^5^. In addition, most useful tools to inform cause of stillbirth have not generally been available in many LMICs. The tools that are traditionally used for assigned cause of stillbirth in LMICs, (i.e., verbal autopsy) do not provide an accurate cause of stillbirth ^6^. Thus, until recently, limited data have been available to inform cause of stillbirths in LMICs.

However, given that most stillbirths occur in LMICs, and because of the increased advocacy for reducing stillbirths in LMICs, determining accurate cause of stillbirth has assumed greater importance ^7^. New tools to evaluate the cause of stillbirth, which are feasible in many LMICs, are now available. These tools include minimally invasive tissue sampling (MITS) – a method using needle biopsies to obtain internal organ tissue from deceased fetuses for histology and pathogen identification ^8– 10^, and multiplex polymerase chain reaction (PCR) to identify a wide range of pathogens in those tissues ^11^. While the ability to study placental histology has been available for some time, the development and publication of the Amsterdam Criteria in 2016 has provided a useful framework to categorize placental lesions ^12^. In addition, to reduce the bias from individual physician observation, many newer studies on stillbirth cause of death have used an independent panel to assess cause of death ^13, 14^.

Given the range of tools now available to inform cause of stillbirth and the limited resources available, we believe the next phase is a determination of which investigations are most informative for stillbirth causation. From a United States’ study of the usefulness of diagnostic tests to determine cause of stillbirth, placental pathology was found to be useful in 64.6% of the cases and fetal autopsy in 42.4%, with other tests far less useful ^15^. Studies from the Netherlands confirm the usefulness of placental pathology in determining COD in the majority of stillbirths ^16^.

More recently, several groups are trying to understand which information, and which specific tests, are useful in determining stillbirth COD in specific LMIC areas ^17– 19^. For the purpose of this exercise, we defined ‘useful’ tests as 1) data that are feasible to obtain accurately and 2) data that help determine a cause of death, or 3) help eliminate a cause of death ^15^. One of the challenges to determine the most informative tests is that for many studies, an expert panel is the final arbiter of the cause of death. The specific information the panel has available can vary by project or case, and it is usually not clear which information individual panel members used to develop their opinion on COD, and how this information was used overall by the panels to designate a specific cause of death. Thus, we have summarized some of the main observations of the authors of this commentary from these panel discussions.

Our first observation is that in these studies conducted in LMIC, even under the best of circumstances, there is usually incomplete information available to panel members. The information may be unavailable due to prohibitive costs, because the technology was unavailable, or because the delivery occurred at home, and as a result the full complement of potentially useful information may not have been available to the panel.

In our view, the full complement of information to determine cause of stillbirth, at best, would include information from several domains ( Table 1). The first domain is maternal clinical history. Useful information in this domain includes a large variety of maternal conditions and especially hypertension, diabetes, and anemia. The second domain includes obstetric conditions that arise during the prenatal period or during labor and delivery including placental abruption, fetal distress, fetal malposition, and uterine rupture. The third domain includes data describing the placenta. These data would include a gross examination, with special emphasis on infarction and hemorrhage, some measures of placental size or weight compared to a reference standard, histology of the placental body, chorioamniotic membranes, and umbilical cord, focusing on signs of inflammation and malperfusion lesions ^20^. The fourth domain, examination of the fetus, first using external observation, includes measurements and weight. Then, using one of several approaches to examine internal organs is important. These approaches may include full diagnostic autopsy, or more recently, MITS, to obtain internal organ tissue samples for histological examination and pathogen PCR for organism identification. We have found it especially useful to present all available data to the panel using a standard computerized approach ^21^.

**Table 1.** Domains of the data considered most useful for cause of stillbirth evaluation.

	Clinical Conditions	Pregnancy conditions	Placental evaluation	Fetal physical and histology evaluation	Polymerase chain reaction (PCR)
**Key** ** Elements**	Hypertension, Diabetes, Anemia	Abruption, Fetal distress, Fetal malposition Uterine rupture	Gross examination Weight Histology (body, membranes umbilical cord) Inflammation, malperfusion lesions Meconium *	Gross examination Fetal weight Lung histology Meconium *	Placenta Lungs Brain/Cerebral spinal fluid Fetal blood
**Source**	Clinical history	Clinical history	Placenta	Physical exam MITS ** or Autopsy	Various tissues

*Meconium seen in any exam was always considered useful **Minimally invasive tissue sampling (MITS)

Our next observation is that some of these data are more useful to the panel members than other data. Determining the usefulness of information is critical since a low-cost and efficient approach is necessary in order for stillbirth COD investigations to become routinely performed. Based on all available data and observations, several types of data will be most useful. The first of these is the relevant maternal clinical and obstetric history. The second is a careful placental evaluation starting with a gross examination including measurement of placental weight (with a comparison to an accepted standard to define small and large placentas), and including histology of the chorioamniotic membranes, umbilical cord and placental body with a focus on inflammation, hemorrhage and malperfusion. The third is an external examination of the fetus, (including weight in comparison to some standard to determine fetal growth restriction) ^22^ and especially for congenital anomalies. While an approach using MITS will likely miss some internal organ anomalies, this outcome is relatively rare.

Finally, we consider potential data from MITS examinations of internal organ histology and PCR for pathogen evaluation of these same tissues and the placenta. Our first observation is that for organ histology, lungs are the most informative organs, while liver and CNS histology provides the least information. Findings of amniotic fluid debris or meconium in the lung, likely due to fetal gasping, is present in somewhat less than half the stillbirths, and often helped the panels determine a diagnosis of fetal asphyxia ^23^. Regarding microbiological analyses, PCR evaluation of blood, CSF, and brain tissue provided the most information ^17^. Microbiological analysis of the placenta and membranes were also informative, as was the finding of meconium on any examination.

In summary, the most common causes of stillbirth in LMICs based on available reports include fetal asphyxia, infection, and congenital anomalies ^24^. In individual cases, the panels used various types of data to choose one or several conditions as the most likely cause(s) of stillbirth. To define the most useful, efficient, and cost-effective data to collect in LMICs to define stillbirth COD, the authors recommend focusing on the clinical history, the placental evaluation, the external examination of the fetus, and when available, fetal tissue evaluation (obtained by MITS) of lung (focused on histology and microbiology) and brain/CSF and fetal blood (focused on microbiological analysis). We recognize that this approach will not identify some causes of stillbirth, including some genetic abnormalities and internal organ anomalies, but we believe it will identify the most common causes of stillbirth, most of the preventable causes ^25^ of stillbirth, and will be the most cost-efficient approach for use in LMICs.

## Data Availability

No data are associated with this article.
