# Supplementary material for: An approach to determining the most common causes of stillbirth in low and middle-income countries: A commentary
Source: Gates Open Res. 2023 Jul 4;7:102. [Version 1] doi: 10.12688/gatesopenres.14112.1 (PMC10547115; doi:10.12688/gatesopenres.14112.1)
Supplement: Peer review history

# Reviewer report: Reviewer response for version 1

**DOI:** 10.21956/gatesopenres.15417.r34116

**Published:** 2023-09-07

**Authors:** Manoja Kumar Das

**Affiliations:** 1 The INCLEN Trust International, New Delhi, Delhi, India

The Open Letter presents the opinion about the most appropriate options for identifying the causes of stillbirth in LMICs.  

 The authors make a relevant point for using a rational approach to the challenging stillbirth issue globally. While there are several methods for stillbirth have been described, the most pragmatic and rational components are yet to be identified and adopted. There is a need to examine the factorial and incremental contribution of the various components including the clinical + gross autopsy/MITS examination + histopathology + microbiology + molecular diagnostic methods to inform the most contextually appropriate combination or bundling. 

 There is a need to clarify the statement "35 by one count", in the sentence, "In high-income countries, cause of death (COD) in stillbirths has been evaluated using several different methods, 35 by one count".

Does the article adequately reference differing views and opinions?

Yes

Are all factual statements correct, and are statements and arguments made adequately supported by citations?

Yes

Is the Open Letter written in accessible language?

Yes

Where applicable, are recommendations and next steps explained clearly for others to follow?

Yes

Is the rationale for the Open Letter provided in sufficient detail?

Yes

Reviewer Expertise:

Public health, infectious diseases, immunization and vaccines.

I confirm that I have read this submission and believe that I have an appropriate level of expertise to confirm that it is of an acceptable scientific standard.

# Reviewer report: Reviewer response for version 1

**DOI:** 10.21956/gatesopenres.15417.r34121

**Published:** 2023-09-07

**Authors:** Lison Rambliere

**Affiliations:** 1 Institut Pasteur, Paris, Île-de-France, France

The article is interesting and easy to read. I thank the authors for their work.

 However, I feel that one essential point is not covered sufficiently. Indeed, as mentioned in the article, these data are very difficult to obtain in certain contexts, notably where deliveries take place at home or with traditional birth attendants, where stillbirths rates are highest. I think it would be interesting to develop this point further, giving ideas or advice for applications in these particularly complicated and poorly documented contexts. How do we deal with the absence of analysis laboratories in certain areas? Or the absence of trained medical staff to carry out these investigations? According to the authors, should the analyses of stillbirth causes be implemented routinely or only in the context of a research protocol?

 I also have a minor comment. Sometimes authors use the abbreviation "LMIC" (e.g.: Stillbirths are one of the most common adverse pregnancy outcomes in low and middle-income countries (LMIC).) and sometimes "LMICs".

Does the article adequately reference differing views and opinions?

Yes

Are all factual statements correct, and are statements and arguments made adequately supported by citations?

Yes

Is the Open Letter written in accessible language?

Yes

Where applicable, are recommendations and next steps explained clearly for others to follow?

Partly

Is the rationale for the Open Letter provided in sufficient detail?

Yes

Reviewer Expertise:

Low and middle income countries, perinatality, children, epidemiology, cause of death, infectious disease, precarity

I confirm that I have read this submission and believe that I have an appropriate level of expertise to confirm that it is of an acceptable scientific standard.
